# Advances in the treatment of erectile dysfunction: what’s new and upcoming?

**DOI:** 10.12688/f1000research.7885.1

**Published:** 2016-03-18

**Authors:** Chintan K. Patel, Nelson Bennett

**Affiliations:** 1 Institute of Urology, Lahey Hospital and Medical Center, Burlington, Massachusetts, USA

**Keywords:** erectile dysfunction, public health, erectile dysfunction treatment

## Abstract

Erectile dysfunction adversely affects up to 20% of all men and is the most commonly treated sexual disorder. The public health implications of this condition are significant and represent a challenge for our healthcare system. The physiological pathways responsible for erections have been extensively studied, and much advancement has been made since the introduction of phosphodiesterase 5 inhibitors. Newer agents, such as dopaminergic and melanocortin receptor agonists, which target central erectogenic pathways, are under investigation. Newer formulations and delivery methods of existing medications such as alprostadil will also be introduced in the near future. Furthermore, low-intensity shockwave lithotripsy and stem cell regenerative techniques are innovative approaches to the treatment of erectile dysfunction.

## Introduction

Erectile dysfunction (ED) is a prevalent condition among men that has significant public health implications, and is defined as the inability to initiate or maintain an erection that is satisfactory for sexual intercourse ^1^. ED is estimated to affect approximately 20% of adult males over the age of 20 ^2^, and by 2025, it is projected to afflict 322 million men worldwide ^3^. ED risk is increased with comorbid conditions such as type II diabetes mellitus (DM), obesity, cardiovascular disease, hypertension, and dyslipidemia ^4^. Interestingly, recent studies have confirmed that ED can serve as a predictor for future cardiovascular disease. In the Prostate Cancer Prevention Trial, the authors reported that men with ED were 45% more likely than men without ED to experience a cardiac event after 5 years of follow up ^5^. ED is well recognized to adversely affect quality of life, decrease work productivity, and increase healthcare costs. Furthermore, it has a considerable financial burden on the public with total expenditures for outpatient management estimated to be $330 million, excluding pharmaceutical costs ^6^. Needless to say, ED is a significant health condition that affects the individual patient and healthcare system as a whole. As such, effective treatment for this condition is paramount.

Currently, there are several treatment options for patients with ED, both non-invasive and invasive. The hallmark of ED treatment has been phosphodiesterase 5 inhibitors (PDE5-Is). These remain the first-line therapy for ED. Currently, there are four PDE5-Is that are FDA approved in the United States: sildenafil (Viagra ^®^), vardenafil (Levitra ^®^), tadalafil (Cialis ^®^), and avanafil (Stendra ^®^), and all have comparable efficacy and side effect profiles. Sildenafil and vardenafil have similar half-lives of 4 hours, while tadalafil has the longest (17.5 hours) and avanafil has the shortest (3 hours). Vardenafil should be used with caution in patients with prolonged QT interval. Second-line therapies include intracavernosal injections with vasogenic agents. These agents can be used alone or in combination and include prostaglandin E _1_, phentolamine, vasoactive intestinal peptide, and papaverine and/or atropine. An alternative second-line therapy consists of intraurethral prostaglandin E1 pellets and vacuum erection devices. These options are invasive, which can be troublesome for patients, and also have side effect profiles. Finally, the most invasive treatment of ED consists of insertion of a penile prosthesis.

Even though there are many treatment options for ED currently, there are still patients who do not respond to or cannot tolerate the above therapies. The focus of this article will not be on the current therapies but rather newer medications and procedures that are currently under investigation in both preclinical and clinical settings for the treatment of ED.

## Non PDE5-I oral agents

Newer pharmacological treatments are focused on targeting alternative pathways in the erectile process, both centrally and peripherally.

***Dopaminergic agents.*** Dopamine operates in the brain as a neurotransmitter and in the periphery it functions like a local messenger. Apomorphine (Uprima) is a dopaminergic agent activating dopamine receptors D1 and D2 at a central level within the paraventricular nucleus of the brain. This medication was first introduced in 1987 by Lal and colleagues ^7^ and has been studied extensively since its debut. It has a rapid onset of action, with a mean time to erection of 12 minutes with approximately 50% efficacy. In the first phase III parallel arm cross-over double-blind study of 854 ED patients, erections occurred rapidly (10–25 minutes) and in 54.4% of attempts at 4 mg (vs. 33.8% placebo), with 50.6% success at intercourse ^8^. This drug achieved regulatory approval in Europe in early 2001, but its use has not been authorized in the United States because of hypotension side effects. Along this same pathway, two dopamine agonists (ABT-724 and ABT-670) selected for the D4 receptor are currently being studied in pre-clinical trials and demonstrated physiologic erections in *in vivo* rat models without the side effects seen with other dopaminergic agents ^9^. Even though ABT-760 and ABT-724 were stopped after phase II trials, the use of similar agents in combination with PDE5-Is appears to be an exciting area of future research.

***Melanocortin receptor agonists.*** Melanocortins are involved in many processes, and their role in controlling sexual function was first reported in the 1960s ^10^. They are linked to the induction of penile erection and the regulation of sexual behavior ^11^. Two well-studied melanocortin receptor agonists are melanotan II and bremelanotide. Early clinical trials showed penile erection in 17 of 20 men who received melanotan II with tip rigidity >80% for an average of 41 minutes ^12^. Side effects reported with melanotan II include nausea, yawning, and a delayed onset of erection (approximately 2 hours). This in turn led to the development of bremelanotide, which can be administered intranasally and has a quicker onset of action. A phase IIB trial with administration of bremelanotide over a 3-month period in patients with DM-induced ED reported significant increases in the International Index of Erectile Function (IIEF) scores ^13^. Intranasal forms of bremelanotide have also shown side effects of nausea and hypertension, and this has led to the development of subcutaneous forms of this therapy. Combination therapy of a subcutaneous melanocortin analogue (PT-141) with sildenafil has been shown to enhance erectile response in a small sample of patients ^14^.

***Soluble guanylate cyclase stimulators and activators.*** PDE5-I efficacy depends on the production of cGMP, which in turn is dependent on nitric oxide (NO) activation of soluble guanylate cyclase (sGC). In some patients, especially post-prostatectomy and DM patients, this pathway is disturbed because of varying amounts of nerve damage ^15^ and the effectiveness of PDE5-Is is reduced significantly. There are two types of compounds that can stimulate sGC: heme-dependent stimulators (BAY 63-2521 and BAY 60-4552) and heme-independent activators (BAY 58-2667). Heme-dependent sGC stimulator functionality depends on the reduced prosthetic heme moiety in the sGC enzyme and synergistic enzyme activation when administered with NO. The activation of sGC by heme-independent activators functions after oxidation or removal of the prosthetic heme group of sGC, highlighting a previously unknown mechanism of enzyme activation. A study using an *in vivo* model using human corpora cavernosal tissue from 16 PDE5-I non-responders found that combination of sGC stimulator and vardenafil enhanced relaxation of the corpora cavernosa ^16^. In this study, human corpora cavernosal tissues were harvested after consent from individuals undergoing penile prosthesis implantation and potent patients undergoing transurethral surgery.

***Rho-kinase inhibitors.*** As mentioned above, endothelial-derived NO plays a critical role in the relaxation of corporal tissue and this pathway is impaired in diabetic patients, which leads to poor erectile function. Phosphorylation of myosin light chain kinase regulates the contraction of smooth muscle in the corpora and dephosphorylation is mediated by smooth muscle myosin phosphatase enzyme. A key regulator of this phosphatase is the serine/threonine kinase Rho-kinase ^17^. Two inhibitors of this Rho-kinase, fasudil and Y-27632, were the first to be studied in rat models, and it was found that relaxation of the corpora was not impaired when subjects were given these medications ^18^. SAR407899 is a more recently developed Rho-kinase inhibitor and has shown promising results in one study when compared to placebo and sildenafil. In this phase II clinical trial, a single dose of SAR407899 was used to assess the increased duration of rigidity of erection. The investigators reported almost double the duration of rigidity (>60%) at the base of the penis with SAR407899 when compared to the placebo group ^19^.

## Topical therapy

Topical therapies are a promising alterative to the current second-line therapies, as they can be safe and easy to use and do not require intraurethral or intracavernosal instrumentation. One of the leading candidates for this type of administration is a medication termed Topiglan. It consists of prostaglandin E1 (alprostadil) combined with SEPA (soft enhancer of percutaneous absorption). Topical alprostadil has been studied in cats and humans and has been shown to induce erectile responses with minimal side effects ^20^. The benefit of this topical therapy is maximized when used as part of a combination regimen such as those including PDE5-Is. This medication has been approved in Canada. Another topical therapy being investigated is topical sildenafil, currently in phase IIa and actively recruiting study participants ^21^. Limitations of this therapy include variable penetration based on individual penile tissue characteristics as well as reported allergic skin reactions.

## Low-intensity shockwave therapy

Extracorporeal low-intensity shockwave therapy (LIST) to the penis has recently emerged as a novel and promising treatment modality for ED. LIST has been previously used to treat a wide variety of urological and non-urological conditions ^22^. The mechanism of action for this treatment consists of sending acoustic waves that generate pressure impulses, which can treat patients with kidney stones, tendinitis, and peripheral vascular disease ^23^. For the treatment of ED ^24^, it is hypothesized that LIST causes cell membrane microtrauma and mechanical stress, which causes an upregulation of angiogenic factors such as vascular endothelial growth factor (VEGF), NO synthase, and von Willebrand factor, which increase angiogenesis and vascularization of tissues ^25^. As such, it is postulated that LIST increases blood flow and endothelial function and results in improvement in erectile function.

Data from initial human trials are promising but are still in the investigational stage ^26^. Gruenwald ^25^ and colleagues performed an open-label, prospective study on patients with severe ED who previously failed PDE5-I therapy. In this group of 29 patients, LIST treatments were administered twice per week for 3 weeks. There was a 3.5-point increase in the IIEF in this patient population, and, furthermore, these men had improved penile hemodynamics and increased blood flow as assessed by plethysmography. The same group of authors more recently published a randomized, double-blinded, sham-controlled study with 58 men ^27^. Significant improvements were again seen in components of the IIEF and penile hemodynamics. More than 50% of patients in the LIST group (vs. none in the sham group) had an erection rigid enough for vaginal penetration. Even though there were no immediate adverse outcomes reported, the true long-term effects of this therapy are yet to be defined. Future studies with longer follow up will be necessary to see if the remodeling of the penile arterial system causes any long-term damage. In summation, current data suggest that LIST is effective in patients with ED and also men who are PDE5-I non-responders. Penile LIST is a novel therapeutic concept and represents another exciting avenue for the treatment of ED.

## Stem cell transplant

Stem cell therapy is a new treatment option that offers the potential to reverse the underlying causes of ED and reduce patient reliance on the transitory effects of PDE5-I medications. It has been studied in several animal models in subjects who poorly respond to PDE5-Is (cavernous nerve injury and DM). Stem cell regenerative therapy is based on the rationale that stem cells can differentiate into a wide variety of cells including endothelial cells, Schwann cells, smooth muscle cells, and neurons ^28^. In ED research, three types of stem cells are commonly used: adipose tissue-derived stem cells, bone marrow-derived stem cells, and muscle-derived stem cells. These can all differentiate into various cell types within the mesodermal germ line. It is hypothesized that multipotent stem cells have beneficial effects on damaged or diseased tissues by releasing various molecular mediators, which lead the host tissue to initiate a regenerative or healing response to diseased or injured tissue responsible for ED. The majority of published studies are based on animal models, but there has been one reported case series of seven men from Korea ^29^. In this study, all diabetic patients, with ages ranging from 57 to 87, were treated with an intracavernosal injection of 15 million allogeneic umbilical cord blood stem cells. Morning erection was regained in six out of the seven men at 6 months from time of injection. With concomitant use of sildenafil, all of these men were able to obtain vaginal penetration. No adverse events were reported. A more recent study reported on a phase I/II clinical trial of intracavernosal autologous bone marrow-mononuclear cells in patients with post-prostatectomy erectile dysfunction ^30^. In the authors’ sample size of 12 patients, they used escalating doses of bone marrow-mononuclear cells and no serious side effects were noted. At 6 months, significant improvements of intercourse satisfaction and erectile function were noted in these patients. These results were preliminary and need to be confirmed in phase II trials. Stem cell transplant therapy is a new frontier in medicine. Larger controlled studies are needed to show any potential benefit at the human level, and further investigation is paramount.

## Gene therapy

Gene therapy is a potential therapeutic option that is another area of investigation for the treatment of ED. Genetic material can be easily injected into the penis, which is advantageous as this direct injection avoids potential systemic complications. Furthermore, the effects of gene therapy are more prolonged in the penis because of a slow turnover rate of the tunica albuginea ^31^. In the first human trial, Melman *et al.* administered a single-dose cavernosal injection of hMaxi-K, a ‘naked’ DNA plasmid carrying the human cDNA encoding the gene for the alpha subunit of the human smooth muscle Maxi-K channel ^32^. No adverse events were noted in the 11 patients who received this therapy. Patients given the two highest doses of hMaxi-K had apparent sustained improvements in erectile function as indicated by improved IIEF domain scores over the length of the study. This was a small study, but the encouraging safety profiles and effectiveness provide evidence that gene therapy is a viable option for the future. The role of stem cell regenerative therapy, in conjunction with gene therapy, will be heavily researched for the treatment of ED in the coming years.

## Conclusion

PDE5-Is have been the cornerstone of ED therapy and because of their effectiveness, the incentive to develop newer drugs has been lacking. Over the past decade, however, we have gained more insight into the molecular and physiologic pathways involving normal erections. This has allowed for the development of new pharmacotherapies for the treatment of ED, especially for patients who are PDE5-I non-responders. Topical alprostadil, shockwave lithotripsy, and stem cell transplants represent innovative treatments and show promise for the next decade. In the future, the treatment of ED will be based on the specific etiologies causing ED, and treatment protocols will be tailored to the particular needs of each individual patient. A larger armamentarium of ED therapies, as summarized by this review, will play a big role in this change, as we will have additional therapies and novel routes of administration that can be offered based on an individual’s specific pathology. Personalized medicine is the future of medicine and will indeed be an important component of ED treatment in the years to come.

## Abbreviations

ED, erectile dysfunction

PDE5-Is, phosphodiesterase 5 inhibitors

DM, diabetes mellitus

sGC, soluble guanylate cyclase

IIEF, International Index of Erectile Function

LIST, low-intensity shockwave lithotripsy

NO, nitric oxide

SEPA, soft enhancer of percutaneous absorption
